# Current perspectives and challenges of using artificial intelligence in immunodeficiencies

**DOI:** 10.1016/j.jaci.2025.06.015

**Published:** 2025-06-28

**Authors:** Jacques G. Rivière, Roser Cantenys-Saba, Gerard Carot-Sans, Jordi Piera-Jiménez, Manish J. Butte, Pere Soler-Palacín, Xiao P. Peng

**Affiliations:** a Universitat Autònoma de Barcelona, Barcelona; b Infection and Immunity in Pediatric Patients Research Group, Vall d’Hebron Institut de Recerca, Barcelona; c Pediatric Infectious Diseases and Immunodeficiencies Unit, Hospital Infantil i de La Dona Vall d’Hebron, Vall d’Hebron Barcelona Hospital Campus, Barcelona; d Jeffrey Modell Diagnostic and Research Center for Primary Immunodeficiencies, Barcelona; e Catalan Health Service, Barcelona, Spain; f Digitalization for the Sustainability of the Healthcare System (DS3) Research Group, Barcelona, Spain; g Division of Immunology, Allergy, and Rheumatology, Department of Pediatrics, University of California, Los Angeles; h Department of Microbiology, Immunology, and Molecular Genetics, University of California, Los Angeles; i Department of Human Genetics, University of California, Los Angeles; j Division of Pediatric Genetic Medicine, Department of Pediatrics, Children’s Hospital at Montefiore, Montefiore Medical Center, Albert Einstein College of Medicine, Bronx; k Department of Genetics, Albert Einstein College of Medicine, Bronx; l New York Center for RDs, Montefiore Medical Center, Albert Einstein College of Medicine, Bronx.

**Keywords:** Artificial intelligence, machine learning, inborn errors of immunity, primary immunodeficiency, secondary immunodeficiency, predictive modeling, genomics, electronic health records, clinical decision support, implementation science, screening algorithm

## Abstract

The rapid growth of artificial intelligence (AI) in health care is promising for screening and early diagnosis in settings that heavily rely on professional expertise, such as rare diseases like inborn errors of immunity (IEI). However, the development of AI algorithms for IEI and other rare diseases faces important challenges such as dataset sizes, availability and harmonization. Similarly, the implementation of AI-based strategies for screening and diagnosis of IEI in real-world scenarios is hampered by multiple factors including stakeholders’ acceptance, ethical and legal constraints, and technologic barriers. Consequently, while the body of literature on AI-based solutions for early diagnosis of IEI continues to expand, clinical utility and widespread implementation remain limited. In this review, we provide an up-to-date comprehensive review of current applications and challenges facing AI use for IEI diagnosis and care.

Primary immune disorders, also known as inborn errors of immunity (IEI) or genetically driven blood and immune disorders, encompass a broad spectrum of genetically and environmentally influenced conditions that compromise immune function.^1–3^ The broad spectrum of their possible clinical presentations often involves complex combinations of infection susceptibility, immune dysregulation, inflammation, atopy, and malignancy risk, posing significant challenges to timely diagnosis. As a result, patients often undergo multiple consultations with various medical specialists before an accurate diagnosis is established. This long journey typically results in a diagnostic delay that may range between 1.3 years and decades, with a 4- to 7-year average in children and longer times in adults in real-world settings.^4–6^ The diagnostic delay in IEI not only exacerbates morbidity and mortality but also increases the burden on health care systems.^7,8^

Traditional diagnostic pathways rely on expert clinical recognition, targeted genetic testing, and functional immunologic assessments, which are time-consuming and costly. Medical settings that depend heavily on professional expertise have been identified as areas that may benefit from artificial intelligence (AI) (see the Online Repository available at www.jacionline.org for a glossary of AI-related terms), which can process vast amounts of clinical and laboratory data, reducing workload and increasing accuracy.^9^ In recent years, a growing body of literature has highlighted promising AI-driven approaches for screening and early diagnosis of diverse disease domains, including IEI.^10^ Fig 1 illustrates the vast amounts of data generated throughout an IEI patient’s journey from symptom onset to definitive diagnosis, highlighting windows of opportunity for AI use at various stages. The availability of machine learning (ML) and deep learning techniques to analyze these data suggests that AI could be a game changer in the management of IEI. However, many issues must be addressed before translating AI-based solutions from research to day-to-day clinical practice.

Here we present a state-of-art overview of AI application on immunodeficiencies, including proof-of-concept examples not only in exploiting electronic health records (EHRs) but also in genomics. Finally, we provide an in-depth analysis of the challenges that may hinder the effective implementation of these strategies in real-world settings.

## CLINICALLY FACING APPLICATIONS OF AI USE IN IMMUNODEFICIENCIES

The potential of AI algorithms for early diagnosis and precise identification of complex and rare diseases (RDs), such as IEI, based on vast amounts of data has raised much interest in both patients and health care professionals. AI-based models potentially improving the management of IEI can be broadly classified into generative and discriminative approaches. Discriminative models focus on classification tasks by learning decision boundaries that separate inputs into predefined categories. Unlike generative models, discriminative algorithms, including support vector machines, logistic regression, and decision trees, require labeled data during training to guide their learning process effectively. In contrast, generative models aim to learn the joint probability distribution of input and output data, allowing them to generate new data samples based on identified patterns.

### Screening and identification of individuals with immunodeficiencies

A major focus of IEI patient advocacy groups has been the achievement of faster and more accessible diagnoses. After suspicion for an IEI arises, the time between a patient’s initial clinical symptoms and referral to a specialist center can be particularly challenging and discouraging for both patients and their families.

However, unlike more common conditions such as diabetes or breast cancer, diagnosing IEI is uniquely complex because it requires distinguishing from among 600+ gene–disease relationships, each presenting with diverse phenotypes. This complexity is further challenged by the limited availability of robust datasets, which are often geographically dispersed and siloed across multiple health care centers and highly diverse EHR systems.

The urgent need to improve diagnostic pathways has spurred awareness campaigns, including the creation of the widely recognized “10 Warning Signs” for IEI^11–14^ or updated versions of them.^15–18^ While these efforts are critical in educating the public and health care providers, they remain insufficient—particularly given the overwhelming amount of information faced by general pediatricians and practitioners across various medical fields.

In this context, supervised ML algorithms have emerged as a promising tool for identifying at-risk patients using EHRs and clinical phenotype databases. In the field of IEI, AI has already demonstrated its value for the first step (eg, identification of patients with potential disease from preexisting databases). An early innovation—the SPIRIT tool—used diagnosis and pharmacy codes within insurance claims data to classify patients into high, medium, or low risk for IEI using a point-based system.^19^ Mayampurath et al^20^ developed a ML model using EHR data to discriminate between potential IEI and control patients for a single center, noting that extension from a logistic regression model to more advanced ML models did not improve performance for their patients, while Rider et al^21^ showed that an ML classifier with weighted rules could screen for IEI across a large population with high accuracy. More recently, ML and data mining techniques were used to identify patients with specific IEI endotypes within the USIDNET registry, while an ML algorithm called PheNet showed ability to identify common variable immunodeficiency patients earlier than traditional methods, with high accuracy in external validation across multiple medical systems.^22^ AI has been used to collect phenotypic data, with natural language processing extracting features from clinical notes^23^ and deep learning imputing missing phenotypes in biobank datasets to expand sample size.^24^ Discriminative and generative facial analysis tools assist clinicians in syndrome identification, with similar strategies applied to histopathology, imaging, and cellular data to generate data-driven models.

Regardless of the algorithm approach, these models have demonstrated the ability to improve IEI screening by leveraging diverse types of nongenomic data, ranging from automated data extraction and application of the classical “10 Warning Signs” to sophisticated deep learning designs for early diagnosis.^11^ Table I lists examples of these approaches.^25–32^

## EXAMPLES FROM OTHER RARE DISEASE DOMAINS

Initiatives in other RD domains have shown promising results with potential for extrapolation to IEIs. For instance, Faviez et al^33^ applied semantic similarity-based approaches to structured and unstructured EHR data to identify patients who may have rare ciliopathies. This approach could be adapted to IEI screening as well.

Retrieval augmentation generation, as demonstrated with RareDxGPT, an enhanced ChatGPT model that integrates information about RDs from an external knowledge resource, has shown potential clinical utility.^34^ This approach could be used to help nonimmunologists improve IEI patient care. However, the primary challenge lies in building and continuously updating the model with a well-curated, high-quality external database.

Recent studies in RD predictive modeling, such as Bayesian approaches in monogenic diabetes,^35^ could inform similar strategies for secondary immune disorders (SIDs) (eg, due to steroids or rituximab). The recalibration methods used in distinguishing maturity-onset diabetes of the young from type 1 diabetes highlight how integrating real-world data into AI algorithms can improve diagnostic precision. Such methods may enhance IEI detection by distinguishing them from the broader and more prevalent SID. However, unlike type 1 diabetes, SID also remains underdiagnosed and poorly characterized, presenting additional challenges for AI application.

At present, although models such as SPIRIT incorporate SIDs to refine risk prediction,^20^ most existing tools explicitly exclude secondary causes.^11,28,29^ Nonetheless, the clinical overlap between primary and secondary immunodeficiencies is well recognized, with shared warning signs and overlapping phenotypes, thus offering an opportunity for mutual detection of both IEI and SIDs.^11,20,22,28,29^ Future models should aim to address this overlap explicitly by incorporating treatment history, medication exposure, and longitudinal immune trends to improve discrimination between IEI and SIDs.

## MOLECULAR DIAGNOSTIC APPLICATIONS OF AI USE IN IMMUNODEFICIENCIES

### AI use for rare genetic disease diagnoses

Significantly reduced sequencing costs have driven the increased clinical adoption of genomics for diagnostic and screening purposes, as well as the growth of large population sequencing initiatives involving greater participant diversity. However, unlike population-based approaches, many datasets for IEIs involve smaller sample sizes and greater complexity than those found in other domains utilizing AI. Additional challenges include the large size of the human haploid genome, which consists of over 3 billion bases, and our still limited understanding of the functional aspects of most genomic variants. For instance, we do not yet know the exact number of protein-coding genes in the human genome, the functions of most of these genes, or the impact of individual variants on gene expression, RNA transcription, protein production, and their ultimate cellular and phenotypic consequences. Moreover, no gene operates in isolation. The ultimate impact of its protein products depends on posttranslational modifications, subcellular trafficking, secretion, and interactions with other proteins and environmental factors. Thus, achieving molecular diagnosis through the identification of a single rare variant driving immune dysfunction is no longer enough. Now we must also confront many layers of additional modulation that determine each individual’s specific expression of disease. Wrestling with this multidimensional complexity offers new opportunities for the application of AI.

### Current AI applications in genomic data processing and analysis

AI-based tools have been used at virtually every stage of genomic analysis (Table II).^36–96^ After sequencing, AI enhances the accuracy of short- and long-read data and aids in alignment-based or alignment-free mapping, crucial for structural variant calling. It also helps create patient- and ancestry-specific reference genomes via *de novo* assembly or pangenome construction. However, the biggest preoccupation of AI applications currently resides with variant calling and prioritization, the processes most directly linked to identifying potential disease-causing loci in sequencing data. Tools like DeepVariant use deep convolutional neural network (CNN)-based approaches to call single nucleotide polymorphisms and small insertions and deletions (indels) from next-generation sequencing data by transforming the variant calling problem into an image recognition task, where the CNN analyzes images of read pileups to predict genotypes.^97^ Many other CNN-based algorithms have been developed to accommodate different sequencing platforms and/or different variants of interest (eg, *de novo,* structural, noncoding, somatic). Many AI-based decision support tools are actively in use for clinical- and research-based variant prioritization. Some are disease or application specific while others are more general; all offer the capacity to aggregate and synthesize multidimensional features including phenotypic, phylogenetic, functional, model organism, and *in silico* pathogenicity predictors (which themselves may be AI based), even for poorly annotated loci. A number of these tools have demonstrated on par^98^ if not superior^80^ discovery and time performance compared with manual analysis or reanalysis^76^ for RDs, though experiences across the IEI realm may vary because of the significant differences in clinical and genetic paradigms between IEI and other RD landscapes.^99^ Some tools apply differential weighting of clinical phenotypes to help refine candidate prioritization. Many of the above programs build in automated variant classification, but stand-alone AI tools also exist for classification of individual variants. Again, the conclusions of these tools often need to be adjusted in the context of gene- and disease-specific knowledge, prompting some to develop more gene-specific tools.

## IMPLEMENTATION CHALLENGES

### Implementation gap

Among the promising applications of large language models, which can process and integrate vast amounts of structured and unstructured data, AI-based screening strategies have emerged as a potential solution to mitigate underdiagnosis in conditions where early detection heavily depends on professional expertise. Some remarkable examples of this use of AI to this purpose include systematic breast cancer screening through mammography,^100^ retinopathy detection from fundoscopy,^101^ and the early identification of atrial fibrillation.^102^

However, while the number of proof-of-concept studies, as well as development and validation efforts for AI-based screening algorithms, including the IEI use cases described in Table I, continues to grow, only a handful of studies have reported the successful implementation of these algorithms in real-world settings and outside the field of IEI.^100,103–105^ Recent experiences in deploying and evaluating AI-based screening tools have highlighted key challenges that contribute to this gap between evidence generation and practical implementation. These challenges span multiple domains, including psychological patient-related factors (eg, privacy concerns, bias), professional considerations (eg, lacking trust in algorithms, need for interpretability), and issues faced by developers and policymakers (eg, data integration, regulatory hurdles).

Notably, all real-world implementations of AI-based screening systems to date have been limited to single centers or a small number of facilities in specialized settings. However, for RD such as IEI, meaningful results are unlikely to be achieved without scaling these algorithms to a nationwide level. Such scaling efforts would inevitably amplify the existing challenges, underscoring the need for robust strategies to address these barriers. Finally, it is worth mentioning that screening for IEI or other RDs at the population level should not be viewed in isolation but rather as part of the broader complexity of health care systems. Primary care physicians, and in general all physicians, are not expected to recognize or retain alerts for every rare condition outside their field, making it essential to develop AI-driven models that prioritize minimizing false alarms. Furthermore, as AI strategies for screening evolve, other areas aside from immunology may also introduce alert systems for early diagnosis. Alert fatigue and its consequences should be addressed in any implementation study and taken into account when assessing statistical power of AI models.^106^ Additionally, in the context of increasing patient involvement in clinical decision-making, the potential for patients to access these alerts through personal health records will need to be thoughtfully considered.

The difficulties of translating evidence-based solutions into real-world practice are not limited to AI. In the past years, increasing awareness has been raised about the need for systematically investigating barriers and facilitators of real-world adoption of evidence-based solutions in medicine. This process, addressed by the emerging field of implementation science, has provided several theory-based implementation frameworks for successful adoption and monitoring of evidence-based solutions in routine care.^107–109^

**FIG 2.**
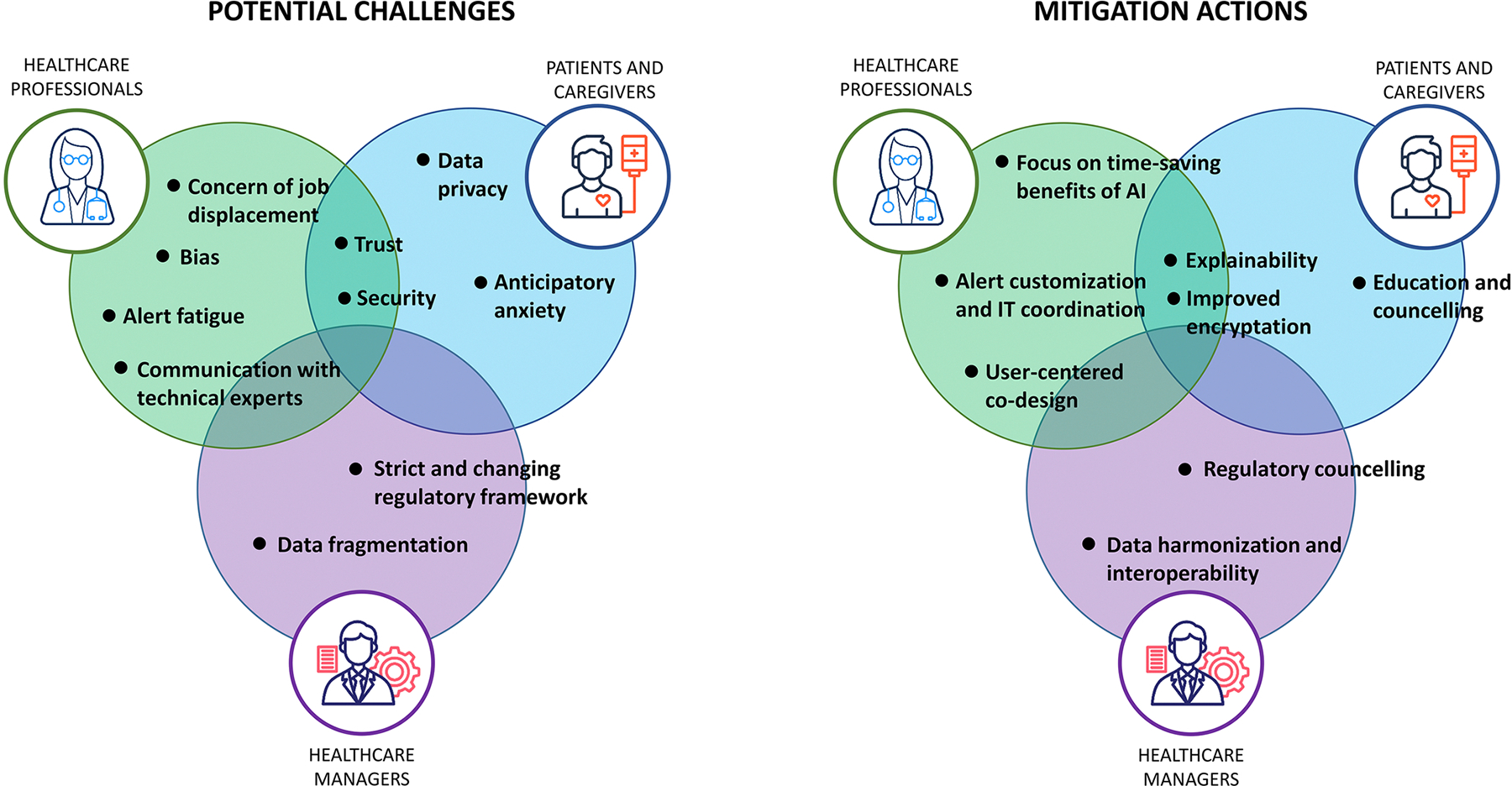
Potential challenges *(left)* and mitigation actions *(right)* associated with implementation of AI-based solutions for early IEI diagnosis.

Despite the promising applications of AI in enabling early diagnosis of IEI, no large-scale AI-driven screening strategies have been effectively implemented to date. Given the complexity of integrating multiple data sources and health care levels to meaningfully identify IEI within the general population, a systematic, theory-based approach to implementation will be essential. The following sections outline some of the most significant potential barriers to this endeavor, analyzed from the perspective of the primary stakeholders: health care professionals, patients and caregivers, and health care systems (Fig 2).

### Regulation in AI development and implementation

The regulatory landscape governing AI development and implementation is continuously evolving. As with many technologic innovations, the establishment of legal and regulatory frameworks struggles to keep pace with rapid advancements in AI-based health care solutions. Currently, AI algorithms designed for clinical decision support are classified as medical devices. Consequently, these algorithms must comply with regulatory standards and obtain clearance from relevant agencies by demonstrating safety, accuracy, and seamless integration with existing health care systems. Given the novelty of AI applications in the medical device sector, the US Food and Drug Administration introduced a specific action plan in 2021 to regulate AI-based software as a medical device.^110^ This action plan was intended to ensure not only the safety and efficacy of approved algorithms but also the robustness of future modifications. A regulatory framework that balances regulatory oversight for safeguarding patient safety and privacy with prevention of excessive regulation that could impede innovation was intended to follow but has not yet been implemented. In the European Union, an overarching framework for AI regulation, the Artificial Intelligence Act, has also recently been developed and began implementation in 2024. However, AI algorithms designed for clinical decision support are categorized as medical devices and must therefore comply with medical device regulation. The European Medicines Agency is currently formulating a specific AI regulatory framework for medical devices, anticipated to take effect by August 2026.^111^

Beyond specific regulatory considerations for AI algorithms to ensure their safe and effective use in health care, their development requires the integration of extensive datasets from diverse sources. Although these datasets undergo deidentification before their use in training and validation, deep learning algorithms have demonstrated the capacity for reidentifying individuals through complex data correlations, including but not limited to genetic information.^112,113^ The potential for patient reidentification poses significant ethical and legal concerns, potentially leading to discrimination in employment and health insurance, as well as emotional distress and diminished trust in the health care system from potential misuse of private medical data.^112,114^ While anonymization techniques exist to mitigate reidentification risks, overly stringent anonymization may compromise data utility, thereby reducing algorithmic accuracy.^115^ Federated learning and swarm learning approaches,^116^ which enable decentralized model training without requiring direct data sharing, represent a promising alternative for facilitating secure data collaboration while preserving patient privacy.^117^

### Stakeholder barriers

Because health care is primarily delivered and received by humans, the adoption of new technologies—regardless of their demonstrated effectiveness—depends on their acceptance by both users (eg, health care professionals) and recipients (eg, patients and caregivers).^118^ In the context of AI, trust in these technologies, particularly among health care professionals, has been identified as a significant barrier to adoption.^9,119^ Survey-based studies indicate that acceptance of AI in health care is heterogeneous and may be influenced by factors such as professional seniority.^9^ However, interpretability has consistently emerged as a key determinant of trust and acceptability among health care professionals.^9,119–121^

A major challenge in achieving explainability is the accuracy toll associated: while advanced deep learning approaches may increase classification performance, their “black box” nature can limit clinical interpretability. In contrast, more transparent models (eg, rule-based or probabilistic frameworks) may be more likely to gain professional acceptance and facilitate integration into clinical workflows, particularly in settings where clinical decision support must be explainable.^121^ Beyond general skepticism associated with novel technologies, explainability (including understanding data sources) is crucial for identifying potential biases,^122^ which can be significant in IEI and be originated from both clinical and genomic information.^123,124^ AI algorithms are inherently trained on datasets that may not comprehensively represent the complexities of health care, thereby perpetuating existing inequities in health care access and delivery.^125,126^ Encouragingly, significant efforts are underway to develop interpretability techniques that mitigate the accuracy–interpretability trade-off.^121^ Recently issued recommendations for increasing transparency and reducing algorithmic bias in AI should also be considered when developing these models.^122^ This guidance is still limited in AI-based genomic tools, which currently rely on general computational tools.^127^ In addition to interpretability and understanding of AI solutions, effective communication between medical and technical experts will also be a mainstay for successful implementations of these solutions. Therefore, user-centered codesign approaches are highly encouraged before planning implementation of evidence-based AI solutions in routine care.^128,129^

Finally, as AI becomes increasingly integrated into medicine, concerns about job displacement among health care professionals cannot be ruled out as a potential barrier to adoption.^119,130^In a screening context, this concern is unlikely to particularly affect specialist physicians, who will continue to oversee the diagnosis of suspected IEI cases. However, it may affect general practitioners, who serve as the first point of contact in identifying individuals at high risk for IEI on the basis of symptoms, medical history, and diagnostic tests. To address these concerns, it has been suggested that AI applications should be limited to tasks where they demonstrably outperform human expertise.^131^ In line with this perspective, organizations such as the American Medical Association have advocated for the term *augmented intelligence* rather than *artificial intelligence* to emphasize AI’s role as an enhancer—rather than a replacement—of human capabilities.^132^

### Data integration and harmonization

One of the fundamental challenges of AI is the vast amount of data required for training and validation. These data must not only be accessible and harmonized but also of high quality to prevent the “garbage in, garbage out” scenario. This need for large datasets is particularly challenging in the context of RDs such as IEI, and even more so when it comes to genetic information, which is not routinely collected in clinical practice in many regions.

The widespread integration of data in professional and personal domains, such as banking and global distribution systems (eg, plane ticketing, hotel bookings, car rentals), has led to the mistaken perception that data integration in medicine is straightforward. However, EHRs are built on an inconsistent blend of vendor-specific models and standards, necessitating complex—and often inefficient—interoperability processes for meaningful data integration and semantic consistency. In research settings, data harmonization can be performed in a controlled way with multiple quality control steps. However, an automated, real-time screening algorithm for identifying and diagnosing patients at high risk of IEI on the basis of information collected throughout the care journey requires true interoperability.

Interoperability is the ability to seamlessly exchange, interpret, and use medical data across different health care providers and systems. This process is hindered not only by regulatory constraints related to data governance and privacy but also by the coexistence of multiple medical ontologies (ie, structured frameworks that define concepts and terms for consistent data representation), such as ICD-10, SNOMED-CT, LOINC (logical observation identifiers names and codes), and DICOM (digital imaging and communications in medicine).^133^

The vast range of medical data sources and formats—including structured clinical data, unstructured information such as clinical notes, and medical imaging—further contributes to data heterogeneity. This may be particularly relevant for genomic data, which are sometimes collected and stored in settings different from routine care. Additionally, many health care facilities—and even individual data-producing devices (eg, a diagnostic imaging machine)—operate on isolated platforms using proprietary information models that are incompatible with hospital information systems, preventing direct access to comprehensive patient data through the EHR. To be effectively utilized, unstructured data—such as radiology reports and pathology findings—require advanced processing techniques. Similarly, vital signs, test results, and EHR entries often differ in format and structure across health care systems.^134^ Without a robust interoperability framework, inefficiencies arise, compromising the quality of care and wasting health care resources.^133^

All these issues related to the codification of health information must be understood in the context of a diverse and heterogeneous world, where health care systems globally are progressing at varying speeds along their digitalization journey.^135^ Beyond the differences between health care systems, the degree of data integration can also vary across different levels of care. Hospitals and specialized settings often have more accessible and integrated data, whereas primary care frequently relies on a fragmented mix of multiple provider organizations or even individual practitioners. In the best-case scenario, this fragmented landscape will likely reduce the real-world effectiveness and generalizability of AI models, increasing the risk of false-positive and -negative results negatives.^136^ In many cases, however, these algorithms may struggle to operate in real-world settings as a result of the unavailability of necessary data at the point of care.

## FUTURE DIRECTIONS

### Emerging applications based on generative approaches

The use of generative AI has grown significantly since the public launch of ChatGPT in November 2022. As previously mentioned, the integration of sharable data and interoperability is key to the successful implementation and scalability of AI use for RDs. For clinical domains like IEI, data curation and standardization are critical but currently very limited. This challenge also presents a unique opportunity for generative AI to play a transformative role.

Beyond assisting health care professionals with routine tasks, generative AI has shown promising results in terms of analyzing EHRs and providing valuable support in coding and patient classification.^137,138^ Additionally, advanced approaches such as generative adversarial networks have demonstrated potential for imputing missing data.^139^ However, in the context of IEI—characterized by complex phenotypes and limited datasets—there is an elevated risk of bias, which must be carefully addressed.

Generative AI can also help bridge language and cultural barriers, thereby improving patient–physician communication, comprehension, and treatment adherence. By facilitating access to medical information in multiple languages with plainer wording, generative AI makes educational materials more inclusive and accessible,^7,8^ reducing reliance on time-consuming manual translations.

Finally, both pre- and posttest counseling are crucial in the genetic testing process. However, health care systems lack enough trained genetics providers to ensure all patients receive proper informed consent counseling before testing and appropriate results counseling afterward for responsible medical use. While our discussion has primarily focused on discriminative models, this is one area where generative AI could be valuable—specifically, using large language model–powered chatbots for patient- and genotype-specific counseling. These chatbots could consider relevant genetic landscapes and inheritance models before testing and provide gene-, disease-, and variant-specific information, along with potential clinical implications, after testing.

### Technologic evolution for AI implementation

As ongoing technologic innovation increases the complexity of health care delivery, several key issues must be addressed to enable successful integration of AI-driven diagnostics into real-time clinical practice. In the long term, a profound transformation of health care information technologies globally will be essential to fully harness AI’s potential for routine screening and diagnosis.

Among existing clinical information systems, openEHR—an open specification for the development of interoperable EHR systems—has been proposed as a vendor-neutral and future-proof solution for clinical data storage. The openEHR standard is a dual-level architecture model (outlined in ISO13606), meaning that it clearly separates information (ie, the data underlying patient records) from knowledge (ie, the rules, guidelines, and domain-specific logic that define how the data is interpreted and used). The information layer is structured through a reference model containing the basic entities for representing any EHR information. The knowledge layer is based on archetypes, which are formal definitions of clinical information models (such as discharge reports, glucose measurements, or family histories) in the form of structured and constrained combinations of reference model entities. The combination of the reference model (representing data entries in the EHR) and the archetype model (organizing these data in a meaningful and usable way) creates systems with powerful evolutionary capabilities as medical practice evolves. Clinical information models may change over time, but the underlying data always remain interoperable—a critical feature for AI integration. Several countries are progressively adopting this standard,^135,140^ while alternative interoperability solutions are being applied in others.^133^ Continuous technologic innovation is increasing the complexity of health care, with diverse settings and devices collecting patient data, making interoperability essential for AI adoption. Health care organizations worldwide are becoming increasingly aware of the need to code and store health information using a unified standard. However, because many health care systems are already in advanced stages of digitalization, implementing such a transformation is expected to be highly challenging.

## CONCLUSIONS

In light of all these different applications and strategies, one might be tempted to undertake a widespread benchmarking comparison to determine the best-performing model. However, this may not be feasible because most of the studies are derived from single centers or databases, which raises concerns about generalizability. Additionally, there is a trade-off between reaching out to every possible source to obtain more detailed information and the associated costs, biases, and privacy issues. As a result, a more pragmatic and scalable approach may be to refine and expand the use of existing models rather than developing new ones to prioritize a context-specific approach to ensure timely real-world implementation.

This mirrors the approach taken with early warning signs in clinical practice: although imperfect, they were widely implemented because their immediate utility outweighs the pursuit of an ideal alternative. Similarly, advancing AI in IEI may depend not on perfection but on optimizing existing models for broader adoption. This requires balancing usability with adaptability, ensuring that models remain flexible enough to accommodate evolving disease paradigms and health care needs. Scientific societies and patient organizations should advocate for real-world deployment, engaging health care decision-makers to prioritize scalability and integration into clinical workflows.

Although still an emerging field, the application of AI to IEI has shown significant promise, both for clinical screening and genomic analysis. However, substantial challenges remain before these powerful tools can be fully integrated into routine clinical practice. Key outstanding challenges remain in terms of data quality, integration, and harmonization; algorithm transparency; regulatory frameworks; data sharing and privacy protection; and equity of representation, access, and implementation. Addressing these concerns is crucial to unlocking the full potential of AI for IEI and other RDs. Moreover, bridging the gap between research and real-world implementation will also require widespread changes in provider practices and health information system architectures.

## Figures and Tables

**FIG 1.**
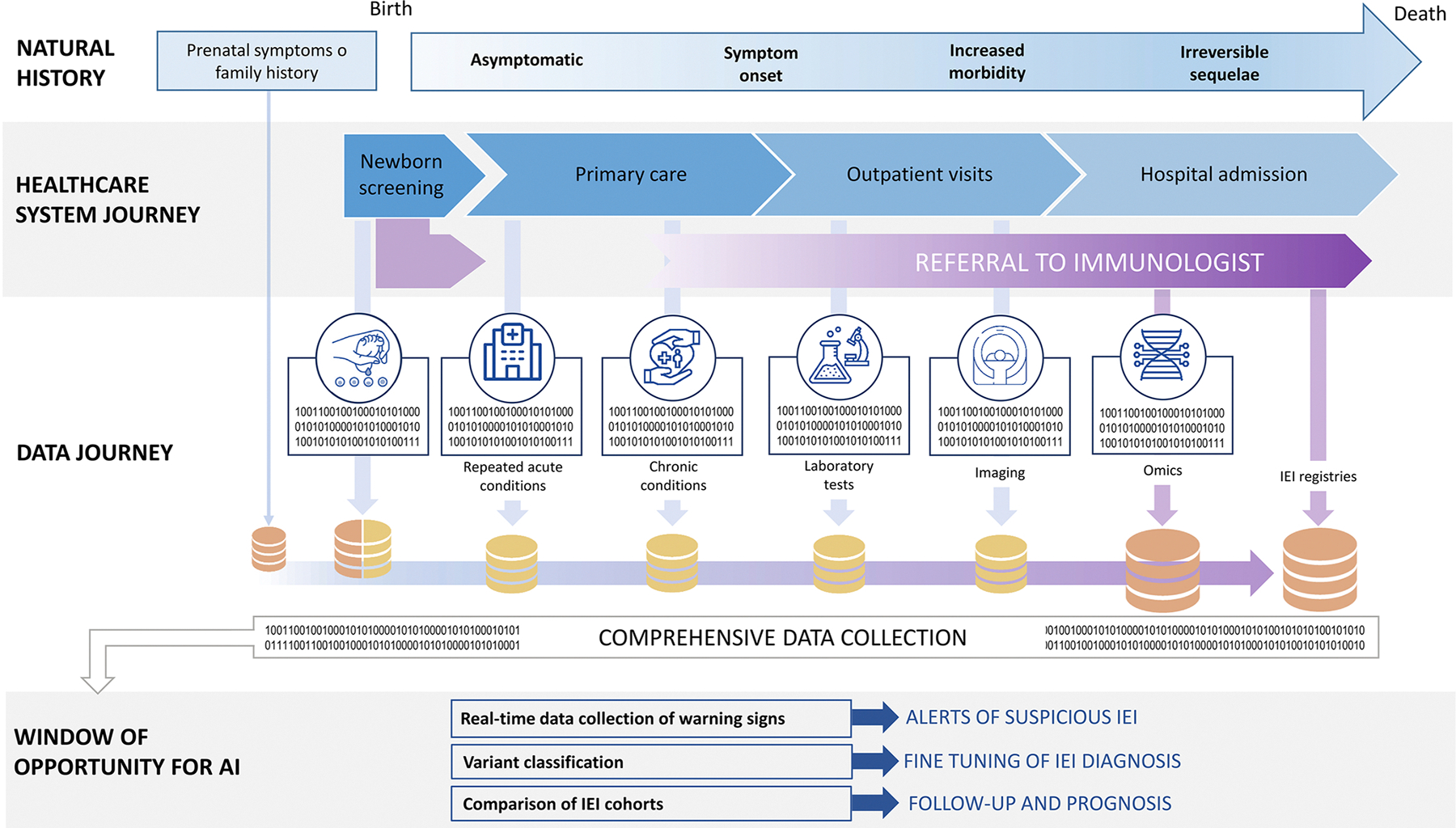
Opportunities for AI in patient and data journey throughout primary immunodeficiencies.

**TABLE I.** ML approaches to diagnosis and/or screening of primary and secondary Immune disorders

Study	Data source	IEI focus	Objectives

Rider (2019–23),^19,21,25^ Modell (SPIRIT) (2017)^26^	Primary and secondary care/structured data	All IEI	Development and validation of AI-driven diagnostic tools for IEI, focusing on predictive modeling and clinical decision support; population screening and early IEI detection using health care claims and electronic records
Roberts (2024)^27^	Secondary care/unstructured data (free text)	All IEI	Proof of concept of free-text analysis in early IEI diagnosis
Johnson (2024)^22^	Secondary care/structured data	CVID/PAD	Identification of CVID by ML using PheNet from EHR
Messelink (2023)^28,29^	Primary care/structured data	PAD	Early diagnosis of PADs in general population and proof of concept of implementation in primary care
Mayampurath (2022)^20^	Secondary care/structured data	PAD*	Risk stratification and prediction model for PADs
Takao (2022)^30^	Secondary care/structured data	All IEI	Development and comparison of risk prediction models to identify children at risk for IEI in immunology clinic
Mendez Barrera (2023)^31^	Registry (USIDINET)/structured data	12 IEI	Proof of concept of computational aid to assist IEI clinical diagnosis
Riviere (2025)^11^	Primary care/structure data (ICD codes)	All IEI	Early diagnosis of IEI in general population and proof of concept of primary care implementation
FitzPatrick (2025)^32^	Secondary care/structure data (ICD codes)	APDS	Identification of APDS patients through ICD codes

*APDS*, Activated p110δ syndrome; *CVID*, common variable immunodeficiency; *ICD*, International Classification of Disease; PAD, predominant antibody deficiency.

**TABLE II.** Examples of AI tools used at different stages of RD diagnostics

Step in clinical diagnostics where AI has been applied	Examples from RD

Identification of patients with potential genetic diseases meriting evaluation from EHR data	• Specific RDs: Fabry,^36^ Pompe,^37^ hereditary transthyretin amyloidosis.^38^ • All RDs: Using diagnostic billing information^39^ or NLP-based feature extraction.^23^
Clinical phenotypic discrimination	• Facial data: PhenoScore,^40^ DeepGestalt.^4^
Laboratory phenotypic discrimination	• Flow and mass cytometry data.^42^ • Cellular imaging data.^43^ • Cancer histopathology data.^44–46^
Refinement of raw sequencing data	• SRS correction: MAC-ErrorReads,^47^ CARE 2.0.^48^ • LRS correction: DeepConsensus for PacBio data,^49^ NanoReviser for ONT data.^50^
Reference genome construction	• *De novo* assembly.^51^ • Pangenome assembly.^52,53^
Reference genome annotation	• Helixer.^54^ • DeepGenGrep.^55^
Mapping to reference genome	• SRS: BWA-MEME,^56^ Embed-Search-Align transformer model.^57^ • LRS: HQAlign,^58^ lordFAST,^59^ kngMap,^60^ S-conLSH.^61^
Variant calling	• SRS and LRS data: Clairvoyante,^62^ HELLO.^63^ • *De novo:* DeNovoCNN.^64^ • SV: Cue.^65^ • Noncoding:^66^ Hi-C interactome data,^67^ genome sequence features 1 chromatin structure,^68^ transfer learning based.^69^ • Somatic: Paired tumor–normal data,^70,71^ tumor-only data.^72^
Variant prioritization and triage	• Phenotype-driven refinement: PhenoApt,^73^ GenomeDiver.^74^ • Decision support tools: Fabric GEM,^75^ Moon,^76^ AI-MARRVEL,^77^ Nostos AION, EvORanker,^78^ eDiVA,^79^ Xrare.^80^ • Pathogenicity prediction: SpliceAI,^81^ AlphaMissense.^82^ • Disease specific: Congenital hearing loss (GenOtoScope),^83^ cancer.^84–86^
Data aggregation platforms with or without variant classification	• General: Franklin Genoox,^87^ VarSome,^88^ LEAP,^89^ MAVERICK,^90^ Genomenon Mastermind.^91^ • Gene or disease specific: *BRCA1/2*,^92^ *ATP7B*,^93^ CPVT- and LQTS-related genes.^94^
Genetic counseling and return of results	• Chatbot for delivery of pretest informed consent counseling.^95^ • Chatbot for return of positive HCPS screening results.^96^

*CPVT*, Catecholaminergic polymorphic ventricular tachycardia; *HCPS*, hereditary cancer predisposition syndrome; *LQTS*, long QT syndrome; *LRS*, long-read sequencing; *NLP*, natural language processing; *SRS*, short-read sequencing.

## Abbreviations used

AI: Artificial intelligence
CNN: Convolutional neural network
EHR: Electronic health record
IEI: Inborn errors of immunity
ML: Machine learning
RD: Rare disease
SID: Secondary immunodeficiency
